# Mapping of seed quality traits in the C genome of *Brassica napus* by using a population carrying genome content of *B. oleracea* and their effect on other traits

**DOI:** 10.1002/tpg2.20078

**Published:** 2021-04-05

**Authors:** Habibur Rahman, Berisso Kebede

**Affiliations:** ^1^ Department of Agricultural, Food and Nutritional Science University of Alberta Edmonton AB T6G 2P5 Canada

## Abstract

Increasing seed oil and protein contents and reducing the content of seed glucosinolates (GSLs) in *Brassica* oilseed crops are important objectives in breeding. By using an oilseed rape (*B. napus* L.) doubled‐haploid (DH) population carrying genome content introgressed from Chinese kale (*B. oleracea* L.), we mapped quantitative trait loci (QTL) for these seed quality traits and investigated their effect on other traits including seed yield. A stable QTL for seed oil content was identified on chromosome C5 at 40–42 Mb position and a QTL for seed GSL content was identified on C9 at 7–8 Mb position. The C5 and C9 QTL alleles for high oil and GSL contents were derived from Chinese kale, demonstrating that high‐oil QTL allele can be found in the parental species of oilseed rape. The low‐GSL QTL allele of C9 exerted a significant positive effect on seed protein content, demonstrating that selection for this QTL allele contributed to higher protein content in canola seed. These two QTL were not affected by field environmental conditions and did not exert a significant effect on days to flowering and seed yield. Thus, the genomic regions and the molecular markers identified in this study should be useful in molecular breeding of the seed quality traits in oilseed rape.

## Core Ideas

- Used a *B. napus* population carrying genome content of *B. oleracea* for mapping seed quality traits.
- Identified a stable QTL on C5 for seed oil and a QTL on C9 for seed GSL content; alleles for high oil and GSL introgressed from *B. oleracea*.
- The low‐GSL allele of C9 exerted a significant positive effect on seed protein content.
- The C5 and C9 QTL did not exert any significant effect on seed yield and days to flowering.

**Abbreviations**
CIM: composite interval mapping
DH: doubled‐haploid
GSL: glucosinolate
GWAS: genome‐wide association study
LOD: logarithm of odds
NIR: near‐infrared
QTL: quantitative trait loci
SNP: single nucleotide polymorphism
SSR: simple sequence repeat

## 1 INTRODUCTION

The demand for canola (*Brassica napus* L. subsp. *napus*) oil in the world market is increasing because of its increasing use as an edible oil as well as for nonedible applications (for review, see Abbadi and Leckband [2011]). Canola seed contain ∼45% oil on whole‐seed basis, and the seed meal remains after oil extraction contain about 36–40% protein on a dry weight basis. The price of canola oil is about three times greater than the price of its seed meal (Canola Council of Canada; https://www.canolacouncil.org); therefore, oil of this crop is, currently, considered as the main product. The amino acid composition of canola meal protein is well balanced, and it is also rich in some of the essential amino acids, such as lysine, methionine and cysteine, which are generally low in most other oilseeds (for review, see Wanasundara, McIntosh, Perera, Withana‐Gamage, and Mitra [2016)). However, the use of this protein in feed and food applications is limited by the presence of several antinutritional components in the seed meal, such as glucosinolate (GSL), fiber, phenolics, phytates, and sinapine (for review, see Khajali and Slominski [2012]); these constituents also impose difficulty in the recovery of this protein from the seed meal through processing methods (for review, see Wanasundara et al., [2016]). Therefore, an increase in seed oil content and reduction of the above‐mentioned antinutritional constituents from the canola seed is desired.

The quantitative trait loci (QTL) mapping of seed oil, protein, and GSL contents in *Brassica* oilseed crops has been carried out by several researchers. Oil content in oilseed rape, or canola, is controlled largely by maternal genotype (Guo et al., 2017) and by a large number of loci from majority of the A and C genome chromosomes with mostly additive effect of the genes (for review, see Rahman, Harwood, & Weselake [2013] and Weselake et al. [2009]); however, epistatic effect and QTL × environment interactions has also been reported for this trait (Delourme et al., 2006; Qiu et al., 2006; Würschum, Maurer, Dreyer, & Reif, 2013; Zhao, Becker, Zhang, Zhang, & Ecke, 2005; Zhao, Dimov, Becker, Ecke, & Möllers, 2008). Quantitative trait loci alleles for high oil content can be dispersed in different canola lines and cultivars, and this can result transgressive segregation in the progeny even when the parents with similar oil content are involved in the cross (Chao et al., 2017; Delourme et al., 2006; Zhao et al., 2005). Therefore, identification of QTL and alleles for high oil content would enable the identification of the parents for crossing and selection of progeny lines with higher oil content. However, the strong negative correlation between seed oil and protein contents (Chao et al., 2017; Grami, Stefansson, & Baker, 1977), which largely arise from colocation of positive and negative alleles controlling these two traits (Chao et al., 2017; Zhao, Becker, Zhang, Zhang, & Ecke, 2006), make it challenging for simultaneous improvement of these two traits. In this regard, identification of independent QTL controlling these two traits (Gül, Becker, & Ecke, 2003; Zhao et al., 2006) would be desired for simultaneous improvement of these two traits.

The QTL mapping of seed GSL content in oilseed rape has also been reported by several researchers (Feng et al., 2012; Gajardo et al., 2015; Howell, Sharpe, & Lydiate, 2003; Toroser, Thormann, Osborn, & Mithen, 1995; Uzunova, Ecke, Weissleder, & Röbbelen, 1995; Zhao & Meng, 2003). The number of QTL detected to date for seed GSL content is far less (five to seven) than the number of QTL reported for seed oil content. Total GSL content of the low GSL oilseed rape cultivars is approximately 12–15 μmol g^−1^ seed; this low GSL content results from homozygous recessive state at all known loci (Rahman, Joersbo, & Poulsen, 2001; Rücker & Röbbelen, 1994). Further reduction of this seed constituent might be possible through identification of additional QTL and alleles for low GSL content.

The growth and development of a plant results from production of photosynthate (sucrose) in the entire green tissue including stem and siliques (source) and allocation to different parts (sink) of the plant such as roots, young shoots, and developing seeds. In the sink cells, sucrose functions as raw material for the production of organic compounds. It often degrades to hexoses and enter into the biosynthesis of cellulase, starch, protein, and lipids (for review see, Wardlaw [1990] and Braun, Wang, & Ruan [2014]), and the genes involved in these various steps including photosynthesis (for review, see Wang, Hendron, and Kelly [2017]) and metabolism of sucrose (for review, see Stein and Granot [2019]) and their networks affects plant growth and development. Thus, it is obvious that the growth of plant parts or plant traits, including accumulation of storage products, is under genetic control (for review, see Johnson and Lenhard [2011] and Gacek, Bartkowiak‐Broda, and Batley [2018]); and colocalized genes and QTL affecting multiple traits (Lv et al., 2016; Shen, Xiang, Xu, Ge, & Li, 2018) or pleiotropic effect of a gene or QTL on multiple traits (Yu et al., 2016) has been reported in *Brassica*. Therefore, it is possible that some of the traits investigated in this study would exert a significant effect on the other traits.

The objective of this research was to identify the QTL affecting seed oil, protein, and GSL content in the C genome of canola and the effect of these QTL on other traits by using a doubled‐haploid (DH) population carrying genome contents introgressed from kale (*B. oleracea* L.). Normally, a biparental DH population is used for QTL mapping through construction of a genetic linkage map, while association analysis is carried out by using a natural population based on historical recombination and linkage disequilibrium between marker and trait, or by using lines derived from multiple crosses. However, a biparental DH population, for example, in barley (*Hordeum vulgare* L.) (Hu et al., 2018), has also shown the potential for mapping different agronomic traits following association mapping approach. In this study, we applied both linkage mapping and genome‐wide association study (GWAS) approaches for identification of environmentally stable QTL affecting the seed quality traits and also investigated their effect on other traits by growing the population in multiple trials over 5 yr.

**Core Ideas**

- Used a *B. napus* population carrying genome content of *B. oleracea* for mapping seed quality traits.
- Identified a stable QTL on C5 for seed oil and a QTL on C9 for seed GSL content; alleles for high oil and GSL introgressed from *B. oleracea*.
- The low‐GSL allele of C9 exerted a significant positive effect on seed protein content.
- The C5 and C9 QTL did not exert any significant effect on seed yield and days to flowering.

## 2 MATERIALS AND METHODS

### 2.1 Plant Materials

A spring growth habit *B. napus* DH population of 94 lines, derived from F_1_ plants of ‘Hi‐Q’ × RIL‐144 (Rahman, Bennett, & Kebede, 2017, 2018), were used in this study. Hi‐Q is a canola cultivar developed by the University of Alberta. The RIL‐144 is an advanced generation zero erucic acid high GSL euploid oilseed rape (2*n* = 38) line developed from Hi‐Q × Chinese kale [*B. oleracea* L. var. *alboglabra* (L. H. Bailey) Musil] (CC, 2*n* = 18) interspecific cross (Rahman, Bennett, Yang, & Thiagarajah, 2011); its C genome carry alleles introgressed from the var. *alboglabra* (Rahman, Bennett, & Séguin‐Swartz, 2015). Based on this pedigree, the DH population of Hi‐Q × RIL‐144 was expected to be segregating primarily for the C genome alleles of var. *alboglabra* and Hi‐Q, and this would allow identification of the QTL for the seed quality traits including the favorable QTL alleles from this genome. In this regard, while considering only the C genome, that is, about half of the whole oilseed rape genome, and segregation for a part of the C genome alleles the use of 94 DH lines for QTL mapping could be justified.

The DH lines together with their parents, Hi‐Q and RIL‐144, were evaluated in nine field trials over 5 yr at South Campus and St. Albert research farms of the University of Alberta, and in a grower's field at Killam and Neapolis, Alberta. Plot size was either 5.0 by 1.3 m or 5.0 by 1.1 m (length by width); number of replications in each trial was two. For convenience of reference, these trials will be designated by the abbreviation of the locations followed by two last digits of the years, for example, SC11 is the trial at South Campus in 2011. Because of the large number of entries in the trials, a modified randomized complete block design was used. In this, each replication was divided into three blocks and the DH lines were randomly nested within these blocks; the parents Hi‐Q and RIL‐144 were included as check in each block. At maturity, the plots were harvested with a plot combine and bulk seeds were used for analysis of seed oil, protein, and GSL contents by near‐infrared (NIR) spectroscopy method (FOSS NIR System Model 6500, Foss North America), and data was reported on a whole‐seed basis at 8.5% moisture. For calibration of the NIR for analysis of oil, protein, and GSL contents, multiple regression equations were developed by the University of Alberta canola laboratory using NIR spectroscopy method. This method is based on the absorption of electromagnetic radiation at wavelength in the range 780–2500 nm. Near‐infrared spectroscopy requires calibration against a reference method for the constituents of interest. Analysis for oil content was based on the principle of nuclear magnetic resonance; protein content was based on total nitrogen content determined following combustion method by using LECO Combustion Nitrogen Determinators (St. Joseph, MI, USA), where nitrogen was converted to protein using a conversion factor of 6.25; GSL reading was based on gas chromatographic determination of trimethylsylyl derivatives of desulphoglucosinolates. All reference methods were approved by the Canadian Grain Commission, Government of Canada (Winnipeg, MB). The calibration curves and NIR equations were also certified by the Canadian Grain Commission.

### 2.2 Molecular marker analysis and linkage map construction

Genomic DNA of the parents and the DH population was extracted from leaf samples using a Sigma DNA extraction Kit (Sigma‐Aldrich) and following manufacturer's instruction. The details of simple sequence repeat (SSR) marker analysis including the source of the markers is reported in Rahman et al. (2017, 2018). Single nucleotide polymorphism (SNP) genotyping of the mapping population was done using the *Brassica* 15K array of Traits Genetics (Seeland, Germany, http://www.traitgenetics.com). Chi‐square test (*p* = .05) for segregation of the SSR and SNP marker alleles was used and the markers showing the expected 1:1 segregation were used for construction of a framework map; markers showing distorted segregation were added later to this map. By using the high‐quality polymorphic SNPs, the bin map was constructed, and the bins included both SNP and SSR markers. The software program JoinMap Version 4.0 (Van Ooijen & Voorrips, 2006, https://www.kyazma.nl/index.php/JoinMap/) was used for linkage analysis and construction of the genetic map using a threshold for goodness of fit ≤5.0, recombination frequency <0.4, and minimum logarithm of odds (LOD) score of 2.0. Assignment of marker order was done using regression mapping algorithm, and map distance (centimorgans, cM) was calculated using Kosambi mapping function (Kosambi, 1944). The details of this linkage map is reported elsewhere (Kebede & Rahman, 2019).

### 2.3 Statistical analysis and QTL mapping

Analysis of variance was carried out based on a linear mixed model using PROC MIXED procedure of SAS version 9.4 (SAS Institute, 2012) and considering the DH lines as fixed effect and location and replication as random effects. The least‐squares mean values of individual trial data was calculated using PDIFF option in SAS, and pooled mean of all trials was calculated based on least‐squares mean values of the individual trials and using the same option. Mean, standard deviation calculated from standard error, and Spearman's correlation coefficient were calculated by using SAS as well. To compare the effect of the alleles of a given QTL on the agronomic and seed quality traits, the DH population was partitioned based on the presence of RIL‐144 or Hi‐Q alleles and were compared for significant difference using TTest procedure of SAS.

Quantitative trait loci mapping was performed using the software program WinQTL cartographer 2.5 (http://statgen.ncsu.edu/qtlcart/WQTLCart.htm) (Wang, Bastern, & Zeng, 2006), where the likelihood of a QTL and its effect was estimated at each centimorgan. The command ‘similarity of loci’ was used to identify the markers that were mapped at the same position of the linkage group (similarity value = 1.000); in this case, only one of these markers was used in QTL mapping. A 1000‐permutation test at *p* < .05 was carried out to determine the genome‐wide LOD threshold value for a QTL. Additive effect and the percentage of the total phenotypic variance explained by the QTL were obtained from the composite interval mapping (CIM) model. Digenic epistasis analysis for additive × additive gene action and epistasis × environment interaction was carried out using the software program QTL Network available at http://ibi.zju.edu.cn/software/qtlnetwork/ (Yang et al., 2008).

A GWAS using the SNP genotypic data and pooled mean phenotypic data of the 94 DH lines was carried out using mixed linear model in the genome association and prediction integrated tool (GAPIT) of the software package R (Lipka et al., 2012). The critical *p*‐value to declare significant association of the marker and trait was set to 1.0 × 10^−3^. The detail of association mapping is described elsewhere (Farid, Yang, Kebede, & Rahman, 2019).

## 3 RESULTS

### 3.1 Variation for traits

Wide variation for all three seed quality traits, oil, protein, and GSL, was found in the DH population as evident from the extent of variation as well as from ANOVA (Supplemental Figures S1–S3; Supplemental Tables S1–S6). ANOVA also showed significant genotype × location interaction for seed oil and GSL contents but not for seed protein content. However, the behavor of the DH lines for these seed quality traits, especially for GSL and oil content, was similar in the field trials as evident from significant correlation (*p* < .05) of the lines in all possible pairs of trials (Supplemental Table S2, S4, and S6). The parent Hi‐Q, on average, contained 45.9% oil and 25.9% protein, which were 4.7% higher and 0.3% lower than the parent RIL‐144 (Supplemental Tables S1 and S3). Seed GSL content of Hi‐Q was 15.3 μmol g^−1^ seed, which was ∼22.7 μmol lower than RIL‐144 (Supplemental Table S5). The DH population showed a continuous variation for seed oil and protein content where most of the lines fell within the range of the two parents for oil content; however, several lines had lower protein than the two parents (Supplemental Figures S1 and S2). In some cases, the distribution was slightly skewed, which may be due to the limited size of the tested population. In case of seed GSL content, the distribution of the population was bimodal within each trial as well as based on the pooled mean across the nine trials (Supplemental Figure S3).

### 3.2 QTL mapping

A total of 1,275 (1,220 SNP and 55 SSR) polymorphic markers were used to construct the nine C genome linkage groups; this map spanned a total length of 814.3 cM and distributed into 270 genetic bins. Of the nine linkage groups, the linkage groups C5 and C9, where the seed quality QTL were detected, spanned a length of 95.0 and 146.8 cM, respectively; the number of markers in a bin of these two linkage groups varied from one to 19, and the distance between the bins in the linkage group varied from 0.2 to 20.6 cM. These two linkage groups could be aligned to the SSR marker‐based linkage map published by Rahman et al. (2017) and the SNP marker‐based map of Clarke et al. (2016).

**FIGURE 1:**
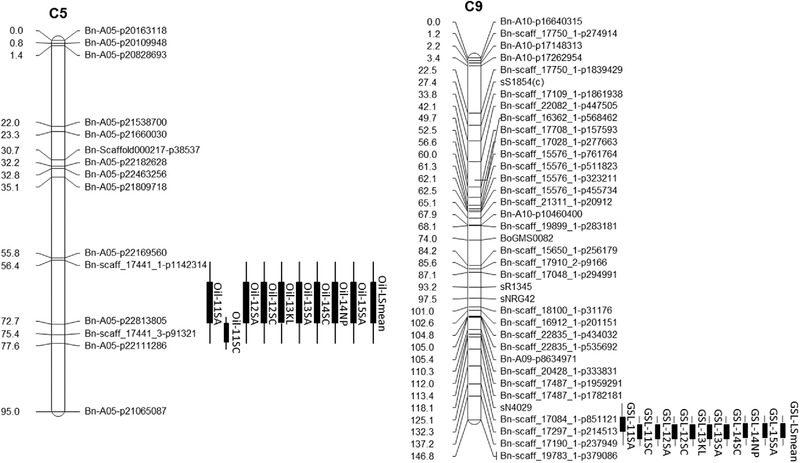
A genetic linkage map of C5 and C9 chromosomes of *Brassica napus* constructed by using a doubled‐haploid population derived from crossing of the *B. napus* parents Hi‐Q and RIL‐144 (carrying C genome content introgressed from *B. oleracea* var. *alboglabra*) and quantitative trait loci mapping of seed oil content on C5 and glucosinolate content on C9

**FIGURE 2:**
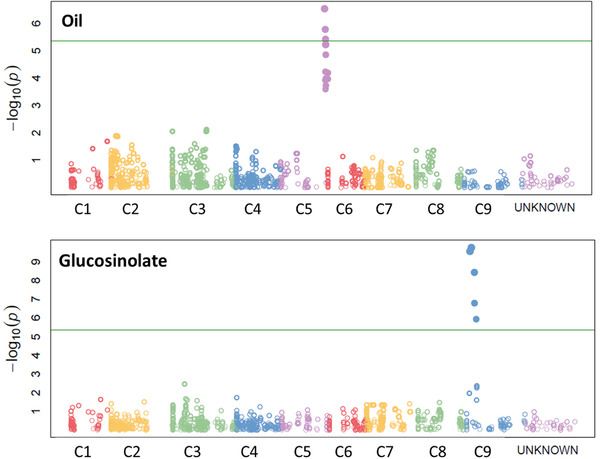
Manhattan plots of genome‐wide association analysis by using SNP markers for seed oil and glucosinolate contents. Solid line indicates the threshold value for declaring a significant association. Association mapping was done by using a doubled‐haploid population derived from crossing of the *Brassica napus* parents Hi‐Q and RIL‐144 (carrying C genome content introgressed from *B. oleracea* var. *alboglabra*)

Composite interval mapping detected a QTL associated with seed oil content on C5 at about 56.4–77.6 cM position (Figure 1) (physical position 40.0–41.7 Mb of the *B. napus* reference genome; Chalhoub et al., 2014). This QTL was detected in all nine trials, mostly with LOD score of >3.0, as well as based on pooled mean data of the nine trials with LOD score of 8.3 (Table 1). This C5 QTL explained 33.6% of the total phenotypic variance and exhibited an additive effect of ∼0.3% oil; the RIL‐144 allele of this QTL contributed increased oil content. By using pooled mean data of the nine trials, this QTL could also be detected through genome‐wide association mapping approach with Bonferroni‐corrected significance threshold −log_10_ (1/[2.40 × 10^−6^]) = 5.6 (Figure 2) (SNP marker position about 40.0–42.9 Mb) (Supplementaal Table S7). In addition to this, 10 genomic regions were identified from six linkage groups through CIM method with LOD score of 1.8‒2.9; however, these QTL could be detected in one or two trials only (Table 1).

**TABLE 1:** Quantitative trait loci (QTL) for seed oil content (percentage of whole seed) in the C genome of *Brassica napus* detected by using a doubled‐haploid (DH) population derived from crossing of *B. napus* parents Hi‐Q and RIL‐144 (carrying C genome content introgressed from *B. oleracea* var. *alboglabra*)

QTL ID^a^	Chromosome	Marker interval	Confidence interval	*R* ^2^ ^b^	TR^2^ ^c^	Additive effect^d^	LOD score^e^
			cM				
Oil‐11SC	C1	sS1867A‐BnGMS9	64.1–72.9	9.1	43.3	0.3	2.3
Oil‐12SA	C1	Bn‐scaff_27039_1‐p129568–Bn‐scaff_20942_1‐p121935	19.5–30.5	7.1	60.8	0.5	2.0
Oil‐13SA	C2	Bn‐scaff_19244_1‐p816342–Bn‐scaff_15838_1‐p1367612	3.4–8.4	11.3	40.1	−0.2	2.5
Oil‐11SA	C2	Bn‐A02‐p13123802–Bn‐A02‐p2364479	47.3–51.9	12.9	35.0	−0.5	2.5
Oil‐12SA	C2	Bn‐A02‐p13123802–Bn‐A05‐p9118240	47.3–53.8	8.3	57.9	−0.1	2.0
Oil‐13SA	C2	Bn‐A02‐p12397585–Bn‐A02‐p12393535	46.5–46.8	13.1	45.8	−0.5	2.6
Oil‐13KL	C2	Bn‐A02‐p19619823–BnFRI4b	58.7–61.8	8.0	53.7	0.5	2.0
Oil‐14SC	C3	Bn‐scaff_24726_1‐p39937–Bn‐scaff_16372_1‐p94487	36.2–36.3	14.0	49.8	−0.6	2.9
Oil‐14NP	C3	Bn‐scaff_24726_1‐p39937–Bn‐scaff_16372_1‐p94487	36.2–36.3	11.9	42.1	−0.7	2.3
Oil‐11SC	C4	Bn‐scaff_23907_1‐p819330–sORH43 (a)	42.9–46.1	9.9	51.5	−0.4	2.0
Oil‐14NP	C4	Bn‐scaff_19248_1‐p66592–Bn‐scaff_18903_1‐p489532	37.0–40.2	8.4	40.5	0.6	2.0
Oil‐11SA	C5	Bn‐scaff_17441_1‐p1142314–Bn‐A05‐p22111286	56.4–77.6	14.9	47.5	−0.3	3.6
Oil‐11SC	C5	Bn‐A05‐p22813805–Bn‐A05‐p22111286	72.7–77.6	8.4	40.5	−0.2	2.0
Oil‐12SA	C5	Bn‐scaff_17441_1‐p1142314–Bn‐A05‐p22111286	56.4–77.6	31.0	53.7	−0.2	7.1
Oil‐12SC	C5	Bn‐scaff_17441_1‐p1142314–Bn‐A05‐p22111286	56.4–77.6	25.1	35.6	−0.2	4.7
Oil‐13KL	C5	Bn‐scaff_17441_1‐p1142314–Bn‐A05‐p22111286	56.4–77.6	17.1	49.9	−0.3	4.5
Oil‐13SA	C5	Bn‐scaff_17441_1‐p1142314–Bn‐A05‐p22111286	56.4–77.6	20.7	40.0	−0.3	4.8
Oil‐14SC	C5	Bn‐scaff_17441_1‐p1142314–Bn‐A05‐p22111286	56.4–77.6	29.3	44.0	−0.5	6.3
Oil‐14NP	C5	Bn‐scaff_17441_1‐p1142314–Bn‐A05‐p22111286	56.4–77.6	25.5	36.9	−0.6	5.3
Oil‐15SA	C5	Bn‐scaff_17441_1‐p1142314–Bn‐A05‐p22111286	56.4–77.6	21.6	44.0	−0.6	5.0
Oil‐11SA	C7	Bn‐scaff_16976_1‐p6292–Bn‐scaff_16200_1‐p226425	2.1–4.3	7.6	48.1	0.4	1.8
Oil‐13SA	C9	Bn‐scaff_17084_1‐p851121–Bn‐scaff_17190_1‐p237949	125.1–137.2	24.8	68.9	0.7	2.0
Oil‐pooled mean^f^	C2	Bn‐A02‐p13123802–Bn‐A05‐p9118240	47.3–53.8	13.8	59.7	−0.7	3.0
Oil‐pooled mean	C3	Bn‐scaff_24726_1–p39937‐Bn‐scaff_16372_1‐p94487	36.2–36.3	7.4	56.9	−0.3	2.1
Oil‐pooled mean	C5	Bn‐scaff_17441_1‐p1142314–Bn‐A05‐p22111286	56.4–77.6	33.6	50.5	−0.3	8.3

^a^ SC, South Campus, University of Alberta; SA, St. Albert, University of Alberta; KL, Killam, Alberta; NP Neapolis, Alberta; ID represents year and location, e.g., SC11 is the trial at South Campus in 2011

^b^ *R* ^2^ is the percentage of phenotypic variance explained by the QTL.

^c^ TR^2^ is the percentage of total phenotypic variance explained by the QTL conditioned on cofactors in composite interval mapping.

^d^ Additive effect is the effect of substitution of Hi‐Q allele by RIL144 allele. Positive value indicates that the allele from Hi‐Q increase seed oil content.

^e^ LOD, logarithm of odds.

^f^ Based on least‐squares mean data of nine trials.

No QTL for seed protein content could be detected consistently in all trials. A total of 15 genomic regions from eight linkage groups were identified; however, in most cases with LOD score <3.0. However, a QTL in the genomic region of the above‐mentoned oil QTL of C5 (56.4–77.6 cM; 40.0–41.7 Mb of the *B. napus* reference genome; Chalhoub et al., 2014) could be detected in four of the nine trials with LOD score of ∼2.0, as well as based on pooled mean data of the nine trials with LOD score of 2.5 (Table 2). This QTL explained ∼10% of the total phenotypic variance and exhibited an additive effect of ∼0.2% protein. As found for the oil QTL, in this case also, the RIL‐144 allele contributed to increased protein content.

**TABLE 2:** Quantitative trait loci (QTL) for seed protein content (percentages of whole seed) in the C genome of *Brassica napus* detected by using a doubled‐haploid (DH) population derived from crossing of *B. napus* parents Hi‐Q and RIL‐144 (carrying C genome content introgressed from *B. oleracea* var. *alboglabra*)

QTL ID^a^	Chromosome	Marker interval	Confidence interval	*R* ^2^ ^b^	TR^2^ ^c^	Additive effect^d^	LOD score^e^
			cM				
Pro‐11SC	C1	sS1867A‐BnGMS9	64.1–72.9	9.3	41.7	−0.2	2.4
Pro‐12SA	C1	sN3754‐sS1867A	58.5–64.1	8.1	42.7	−0.3	2.2
Pro‐11SC	C2	Bn‐scaff_19244_1‐p816342–Bn‐scaff_15838_1‐p1367612	3.4–8.4	15.9	46.8	−0.3	2.6
Pro‐12SA	C2	Bn‐scaff_19244_1‐p816342–Bn‐scaff_15838_1‐p1367612	3.4–8.4	14.5	41.6	−0.4	3.0
Pro‐11SA	C2	Bn‐A02‐p13123802–Bn‐A02‐p2364479	47.3–51.9	14.6	47.0	0.4	3.2
Pro‐14SC	C3	Bn‐scaff_18849_1‐p209120–Bn‐scaff_16372_1‐p94487	33.4–36.3	11.6	42.1	0.1	2.5
Pro‐12SC	C4	Bn‐scaff_27513_1‐p62675–Bn‐scaff_16888_1‐p428653	58.9–61.3	15.9	48.0	0.4	2.5
Pro‐15SA	C4	Bn‐scaff_27513_1‐p62675–Bn‐scaff_16888_1‐p428653	58.9–61.3	9.9	48.6	0.4	3.4
Pro‐12SC	C4	Bn‐scaff_16876_1‐p257162–Bn‐scaff_19208_1‐p130237	77.3–78.5	13.1	43.0	0.4	2.3
Pro‐12SA	C5	Bn‐A05‐p20828693–Bn‐Scaffold000217‐p38537	1.4–30.7	12.6	45.9	−0.3	2.6
Pro‐11SA	C5	Bn‐scaff_17441_1‐p1142314–Bn‐A05‐p22813805	56.4–72.7	15.8	57.3	−0.4	2.1
Pro‐11SC	C5	Bn‐scaff_17441_3‐p91321–Bn‐A05‐p22111286	75.4–77.6	7.5	42.9	−0.2	1.9
Pro‐12SC	C5	Bn‐scaff_17441_3‐p91321–Bn‐A05‐p22111286	75.4–77.6	9.4	39.2	−0.3	2.3
Pro‐14NP	C5	Bn‐scaff_17441_1‐p1142314–Bn‐scaff_17441_3‐p91321	56.4–75.4	7.3	35.9	0.4	1.9
Pro‐11SA	C6	Bn‐scaff_18206_1‐p457621–Bn‐scaff_15763_1‐p604295	28.2–39.1	9.2	47.6	−0.1	2.3
Pro‐12SA	C6	Bn‐scaff_15763_1‐p233999–Bn‐scaff_15763_1‐p604295	37.9–39.1	8.5	44.8	−0.3	2.0
Pro‐14NP	C6	Bn‐scaff_18206_1‐p457621–Bn‐scaff_15763_1‐p604295	28.2–39.1	8.9	34.4	−0.1	2.1
Pro‐11SC	C6	sR0282R‐BnGMS151	87.4–89.3	6.3	41.4	0.2	1.9
Pro‐12SC	C7	Bn‐scaff_16976_1‐p6292–Bn‐scaff_16200_1‐p226425	2.1–4.3	10.8	40.4	0.3	2.7
Pro‐11SA	C7	Bn‐scaff_16976_1‐p6292–Bn‐scaff_16200_1‐p226425	2.1–4.3	9.8	48.9	−0.3	2.4
Pro‐15SA	C9	Bn‐scaff_18100_1‐p31176–Bn‐scaff_16912_1‐p201151	101.1–102.6	33.6	82.4	0.2	2.2
Pro‐13KL	C9	Bn‐scaff_17084_1‐p851121–Bn‐scaff_17190_1‐p237949	125.1–137.2	19.5	38.2	0.4	2.0
Pro‐15SA	C9	Bn‐scaff_17297_1‐p214513–Bn‐scaff_17190_1‐p237949	132.3–137.2	17.7	49.7	0.1	3.7
Pro‐pooled mean^f^	C5	Bn‐scaff_17441_1‐p1142314–Bn‐A05‐p22111286	56.4–77.6	10.0	45.0	−0.2	2.5
Pro‐pooled mean	C9	Bn‐scaff_17297_1‐p214513–Bn‐scaff_17190_1‐p237949	132.3–137.2	19.1	42.1	0.1	3.8

^a^ SC, South Campus, University of Alberta; SA, St. Albert, University of Alberta; KL, Killam, Alberta; NP Neapolis, Alberta; ID represents year and location, e.g., SC11 is the trial at South Campus in 2011

^b^ *R* ^2^ is the percentage of phenotypic variance explained by the QTL.

^c^ TR^2^ is the percentage of total phenotypic variance explained by the QTL conditioned on cofactors in composite interval mapping.

^d^ Additive effect is the effect of substitution of Hi‐Q allele by RIL144 allele. Positive value indicates that the allele from Hi‐Q increase seed protein content.

^e^ LOD, logarithm of odds.

^f^ Based on least‐squares mean data of nine trials.

In case of seed GSL content, a QTL was detected on C9 at about 118.1‒137.2 cM position (Figure 1) (physical position 7.0–8.6 Mb of the *B. napus* reference genome; Chalhoub et al., 2014) in all nine trials with LOD score of 8.4‒26.8, as well as based on pooled mean data of the nine trials with LOD score of 21.8 (Table 3). This QTL explained ∼73.8% of the total phenotypic variance and exhibited an additive effect of ∼2.8 μmol GSL g^−1^ seed. The high GSL allele of this QTL originated from RIL‐144. This QTL could also be detected through genome‐wide association mapping approach with Bonferroni‐corrected significance threshold −log_10_ (1/[1.14 × 10^−6^]) = 5.9 (Figure 2) (SNP marker position 7.0–8.6 Mb) (Supplemental Table S8). In addition to this, 10 genomic regions from six linkage groups were identified, each in one to three trials, through CIM method with LOD score of 2.0‒4.4 (Table 3).

**TABLE 3:** Quantitative trait loci (QTL) for seed glucosinolate content (μmol g^−1^ seed) in the C genome of *Brassica napus* detected by using a doubled‐haploid (DH) population derived from crossing of *B. napus* parents Hi‐Q and RIL‐144 (carrying C genome content introgressed from *B. oleracea* var. *alboglabra*)

QTL ID^a^	Chromosome	Marker interval	Confidence interval	*R* ^2^ ^b^	TR^2^ ^c^	Additive effect^d^	LOD score^e^
			cM				
GSL‐14SC	C1	Bn‐scaff_19244_1‐p816342–Bn‐scaff_15838_1‐p1804241	3.4–13.1	5.4	73.2	−2.2	2.8
GSL‐15SA	C1	Bn‐scaff_19244_1‐p816342–Bn‐scaff_15838_1‐p1804241	3.4–13.1	3.8	75.0	−1.9	2.3
GSL‐12SA	C1	Bn‐scaff_27039_1‐p129568–Bn‐scaff_20942_1‐p121935	19.5–30.5	2.7	83.9	1.8	2.0
GSL‐11SC	C1	Bn‐scaff_20942_1‐p121935–BrEST‐64	30.5–34.1	4.0	77.7	2.1	2.2
GSL‐15SA	C4	Bn‐scaff_15908_1‐p854571–Bn‐scaff_15908_1‐p1124457	32.2–34.6	2.9	74.5	2.2	1.9
GSL‐14NP	C4	Bn‐scaff_19248_1‐p66592–Bn‐scaff_23907_1‐p819330	37.0–42.9	4.0	72.7	2.3	1.9
GSL‐15SA	C4	Bn‐scaff_19248_1‐p66592–Bn‐scaff_23907_1‐p819330	37.0–42.9	3.6	75.2	2.4	2.2
GSL‐14SC	C4	Bn‐scaff_19043_1‐p83155–Bn‐scaff_19208_1‐p527194	69.9–71.5	4.0	72.6	2.7	2.2
GSL‐14SC	C4	Bn‐scaff_16876_1‐p1114982–Bn‐scaff_19208_1‐p130237	76.4–78.5	4.1	72.7	2.7	2.3
GSL‐13SA	UL‐C5^f^	sS2131a–sNRF57	32.2–54.3	27.8	79.5	−5.9	3.2
GSL‐13KL	UL‐C5	sS2131a–sNRF57	32.2–54.3	31.0	77.9	−5.6	4.4
GSL‐14NP	UL‐C5	sS2131a–sNRF57	32.2–54.3	11.4	75.3	−4.7	4.0
GSL‐12SA	UL‐C5	sS1854c–sS1940b	189.5–212.4	9.3	89.4	−6.6	2.7
GSL‐11SC	C6	Bn‐scaff_18206_1‐p457621–Bn‐scaff_15763_1‐p233999	28.2–37.9	3.7	76.5	2.5	2.0
GSL‐14SC	C8	Bn‐scaff_21786_1‐p111311–Bn‐scaff_16770_1‐p3009638	23.2–44.1	5.9	73.7	−2.7	2.8
GSL‐14NP	C8	Bn‐scaff_21786_1‐p111311–Bn‐scaff_16174_1‐p1255232	23.2–44.9	8.7	73.0	−4.3	4.2
GSL‐15SA	C8	Bn‐scaff_16231_1‐p850157–Bn‐scaff_16231_1‐p792817	34.1–35.2	3.9	75.8	−2.9	2.4
GSL‐11SA	C9	sN4029‐Bn‐scaff_17190_1‐p237949	118.1–137.2	52.6	86.2	−3.2	15.3
GSL‐11SC	C9	Bn‐scaff_17084_1‐p851121–Bn‐scaff_17190_1‐p237949	125.1–137.2	67.2	79.9	−2.6	16.3
GSL‐12SA	C9	Bn‐scaff_17084_1‐p851121–Bn‐scaff_17190_1‐p237949	125.1–137.2	82.3	89.3	−2.7	26.8
GSL‐12SC	C9	Bn‐scaff_17084_1‐p851121–Bn‐scaff_17190_1‐p237949	125.1–137.2	62.5	87.1	−2.2	23.1
GSL‐13KL	C9	Bn‐scaff_17084_1‐p851121–Bn‐scaff_17190_1‐p237949	125.1–137.2	42.7	63.6	−2.6	8.4
GSL‐13SA	C9	Bn‐scaff_17084_1‐p851121–Bn‐scaff_17190_1‐p237949	125.1–137.2	40.3	61.8	−2.3	7.9
GSL‐14SC	C9	Bn‐scaff_17297_1‐p214513–Bn‐scaff_17190_1‐p237949	132.3–137.2	60.5	79.2	−2.5	17.0
GSL‐14NP	C9	Bn‐scaff_17297_1‐p214513–Bn‐scaff_17190_1‐p237949	132.3–137.2	67.1	80.3	−2.8	17.3
GSL‐15SA	C9	Bn‐scaff_17084_1‐p851121–Bn‐scaff_17190_1‐p237949	125.1–137.2	64.6	82.9	−3.7	19.8
GSL‐pooled mean^g^	C4	Bn‐scaff_23907_1‐p807570–Bn‐scaff_23907_1‐p819330	41.6–42.9	3.0	82.4	0.4	2.3
GSL‐pooled mean	UL‐C5	sS2131a‐sNRF57	32.2–54.3	4.7	80.9	−2.4	2.7
GSL‐pooled mean	C8	Bn‐scaff_16231_1‐p850157–Bn‐scaff_16231_1‐p792817	34.1–35.2	2.9	78.0	−1.6	1.9
GSL‐pooled mean	C9	Bn‐scaff_22835_1‐p434032–Bn‐A09‐p8634971	104.8–105.4	2.5	80.0	−1.6	2.0
GSL‐pooled mean	C9	Bn‐scaff_17084_1‐p851121–Bn‐scaff_17190_1‐p237949	125.1–137.2	73.8	85.6	−2.8	21.8

^a^ SC, South Campus, University of Alberta; SA, St. Albert, University of Alberta; KL, Killam, Alberta; NP Neapolis, Alberta; ID represents year and location, e.g., SC11 is the trial at South Campus in 2011

^b^ *R* ^2^ is the percentage of phenotypic variance explained by the QTL.

^c^ TR^2^ is the percentage of total phenotypic variance explained by the QTL conditioned on cofactors in composite interval mapping.

^d^ Additive effect is the effect of substitution of Hi‐Q allele by RIL144 allele. Positive value indicates that the allele from Hi‐Q increase seed glucosinolate content.

^e^ LOD, logarithm of odds.

^f^ Unlinked linkage group; however, C5 markers found.

^g^ Based on least‐squares mean data of nine trials.

### 3.3 Effect of the C5 and C9 QTL

**FIGURE 3:**
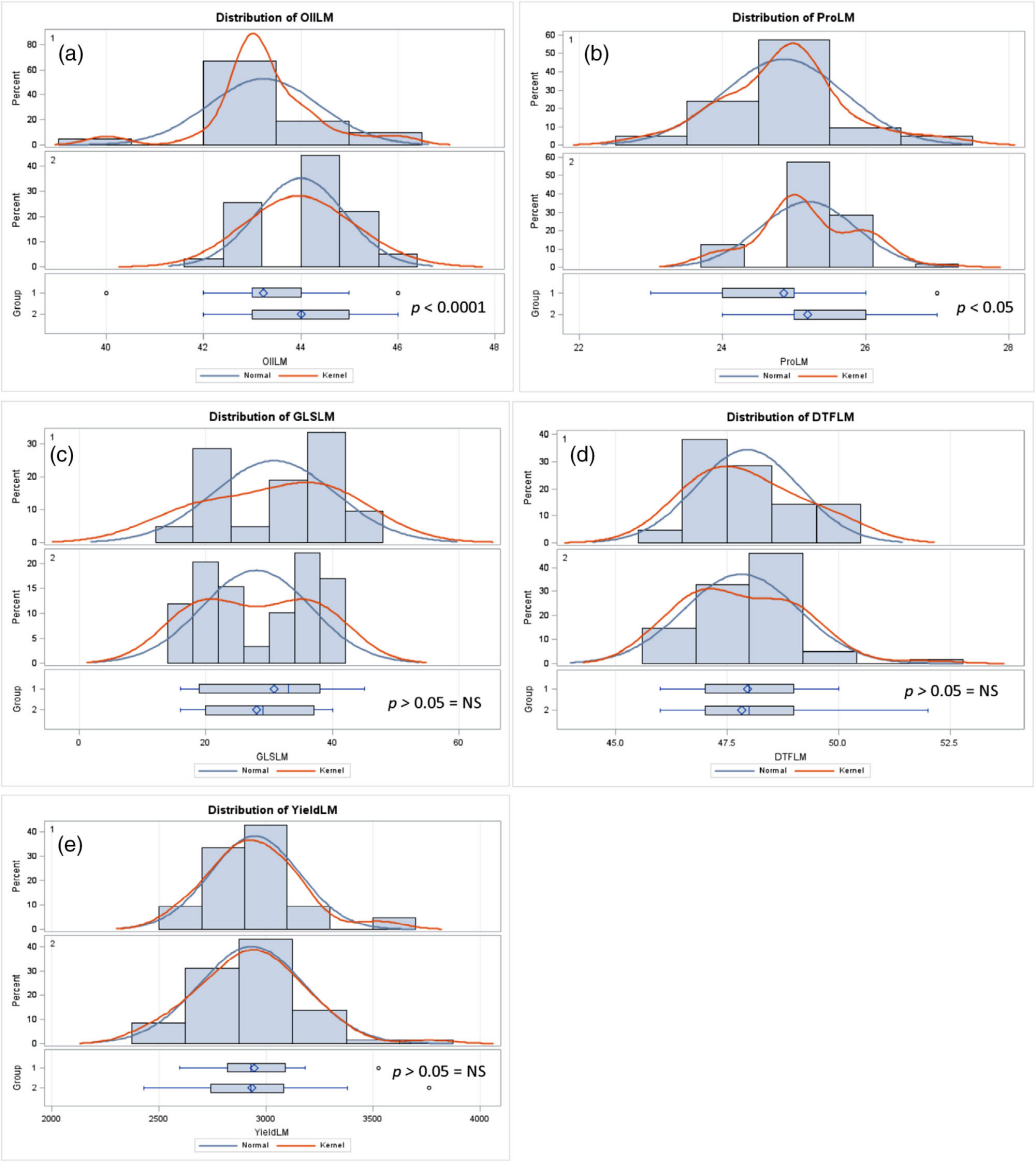
Effect of the C5 QTL alleles of Hi‐Q and RIL‐144 (carrying C genome content introgressed from *Brassica oleracea* var. *alboglabra*) of *B. napus* in a doubled‐haploid (DH) population derived from Hi‐Q × RIL‐144 on seed quality and agronomic traits including seed yield. The DH population tested in nine yield trials in 2011–2015 in Alberta and partitioned for the quantitative trait loci alleles resulting 21 lines with Hi‐Q‐allele and 59 lines with RIL‐144‐allele. (a) Oil content (percentage of whole seed), (b) protein content (percentage of whole seed), (c) glucosinolate content (μmol g^−1^ seed), (d) days to flowering, (e) seed yield (kg ha^−1^). The upper and lower part of the Figure shows frequency distribution of the DH lines differing for Hi‐Q and RIL‐144 alleles, respectively, that is, the effect of these alleles on different traits. The vertical line inside the box plot indicates the median; diamond indicates the mean (middle position of the diamond) and the upper and lower 95% of the mean; whiskers indicate the variability outside the upper and lower quartiles; and the circles beyond the whiskers indicates the outliers

**FIGURE 4:**
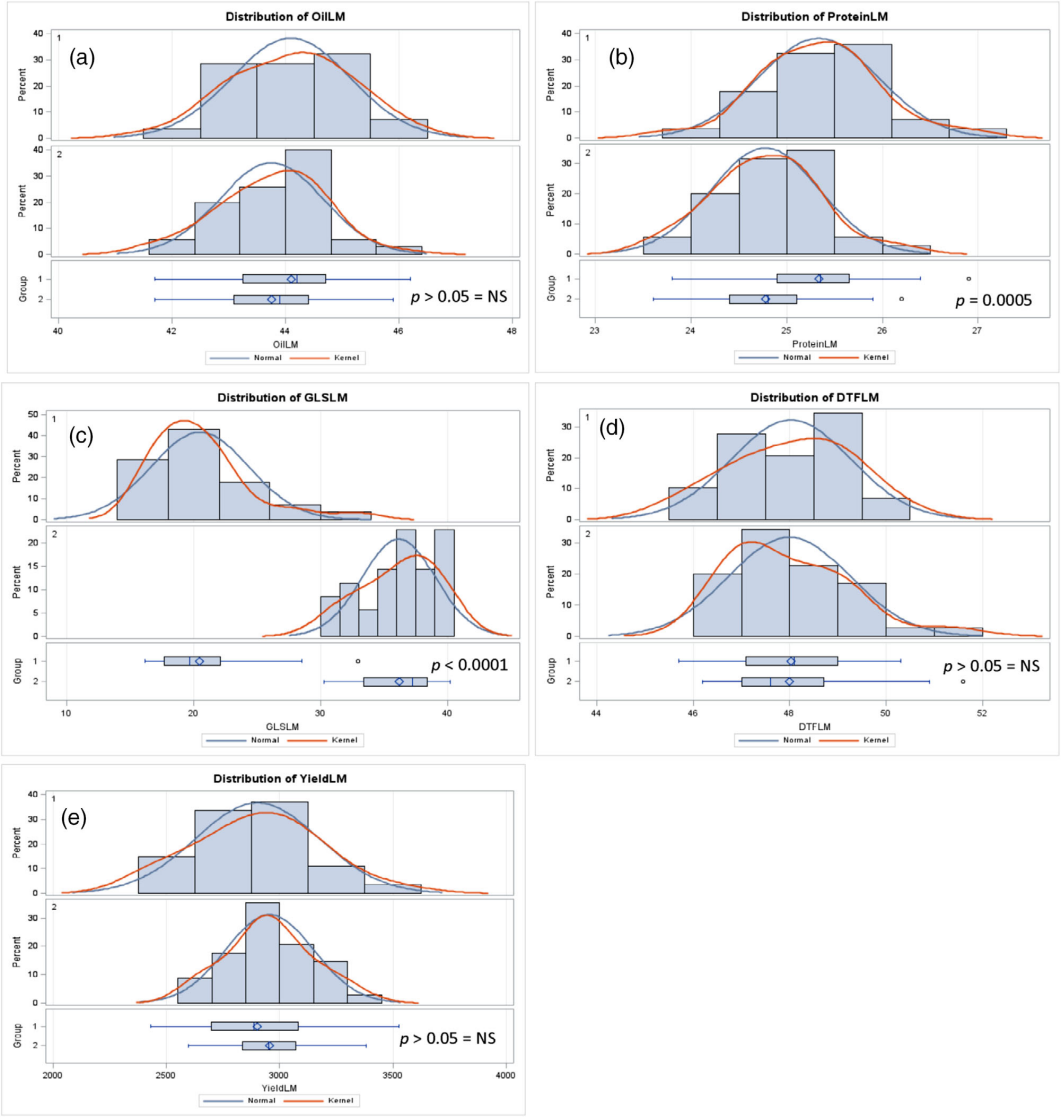
Effect of the C9 QTL alleles of Hi‐Q and RIL‐144 (carrying the C genome content introgressed from *Brassica oleracea* var. *alboglabra*) of *B. napus* in a doubled‐haploid (DH) population derived from Hi‐Q × RIL‐144 on seed quality and agronomic traits including seed yield. The DH population tested in nine yield trials in 2011–2015 in Alberta and partitioned for the quantitative trait loci alleles resulting 28 lines with Hi‐Q‐allele and 38 lines with RIL‐144‐allele. (a) Oil content (percentage of whole seed), (b) protein content (percentage of whole seed), (c) glucosinolate content (μmol g^−1^ seed), (d) days to flowering, (e) seed yield (kg ha^−1^). The upper and lower part of the Figure shows frequency distribution of the DH lines differing for Hi‐Q and RIL‐144 alleles, respectively, that is, the effect of these alleles on different traits. The vertical line inside the box plot indicates the median; diamond indicates the mean (middle position of the diamond) and the upper and lower 95% of the mean; whiskers indicate the variability outside the upper and lower quartiles, and the circles beyond the whiskers indicates the outliers

To further confirm the effect of the C5 QTL on seed oil content as well as its possible effect on other seed quality and agronomic traits including seed yield, the DH population was partitioned into two groups based on the QTL flanking markers of Hi‐Q vs. RIL‐144 and were tested for significant difference. Similar approach was taken to understand the effect of the C9 QTL alleles. In case of the C5 QTL, a significant difference was found between these two groups (*t* = 7.40; *p* < .0001) for seed oil content, where RIL‐144 allele in homozygous condition increased ∼1% oil (Figure 3; Supplemental Table S9); this QTL also exerted significant positive effect on seed protein content (*t* = 2.17; *p* = .035). In case of the C9 QTL, a strong significant effect of the RIL‐144 allele was found on seed GSL content (*t* = 18.50; *p* < .0001) (Figure 4); this allele contributed ∼15.6 μmol GSL g^−1^ seed in homozygous condition (Supplemental Table S9). This QTL also exerted a significant effect (*t* = 3.69; *p* < .0005) on seed protein content where the low‐GSL allele of Hi‐Q increased ∼0.5% protein. None of these two QTL exerted any significant effect on days to flowering and seed yield (Figures 3 and 4; Supplemental Table S9).

## 4 DISCUSSION

Quantitative trait loci mapping of seed oil content in oilseed rape has been conducted by several researchers: Burns et al. (2003), Chao et al. (2017), Chen et al. (2010), Chen et al. (2013), Delourme et al. (2006), Ecke, Uzunova, and Wcißleder (1995), Fu et al. (2017), Gajardo et al. (2015), Javed et al. (2016), Jiang et al. (2014), Körber et al. (2016), Liu et al. (2016b), Qiu et al. (2006), Schatzki, Ecke, Becker, and Möllers (2014), Sun et al. (2012), Teh and Möllers (2016), Wang et al. (2013), Würschum, Liu, Maurer, Abel, and Reif (2012), Yu et al. (2016), and Zhao et al. (2005, 2006, 2008, 2012). Based on these studies, all nine C genome chromosomes carry QTL for this trait. Additive effect of the individual QTL alleles in most of the studies varied from 0.2–1.0% and individual QTL explained 2–20% of the total phenotypic variance. A large number of the seed oil QTL has also been found to be influenced by environment (e.g. Jiang et al., 2014; Yu et al., 2016; Zhao et al., 2005). However, in the present study, by using a population carrying genome content of *B. oleracea*, we detected a QTL on C5 that could be detected in all nine field trials conducted over 5 yr, indicating its high stability across the field environments. Based on the QTL marker alleles, it can be inferred that the high‐oil allele has been introgressed from *B. oleracea*; in this regard, it could be a novel QTL in canola. Several researchers, such as Chao et al. (2017), Javed et al. (2016), Körber et al. (2016), Sun et al. (2012), and Wang et al. (2013), also detected seed oil QTL on C5; however, in most cases, these QTL could not be detected in all field trials. In contrast, Delourme et al. (2006) reported a QTL on C5 in all three trials while working with a winter canola population; however, this QTL could not be aligned to our linkage map because of a lack of a common marker. Körber et al. (2016) detected a seed oil QTL on C5 at 9.49‐Mb position in the *B. napus* genome; in contrast, the QTL that we detected in the present study is located at 40.0–42.9 Mb position of C5 (Supplemental Table S7). Thus, the results may suggest that a QTL for seed oil content has been introgressed from *B. oleracea* into *B. napus*. It is also probable that this allele has been created from a novel recombination as has been reported by Schranz and Osborn (2000) for flowering time allele in resynthesized *B. napus*; further investigation would be needed to confirm this. However, this QTL has not been affected by the field environmental conditions in Alberta and did not exert any effect on days to flowering and seed yield; thus, this QTL can reliably be used for increasing seed oil content in canola.

The C5 QTL of oil content seems to be colocalized with a QTL for seed protein content. Interestingly, the QTL allele for high seed protein content also derived from *B. oleracea*; however, this QTL was sensitive to the environment. The occurrence of the positive alleles for both oil and protein contents in the same genomic region contradicts the results reported by several researchers. For example, Zhao et al. (2006) and Chao et al. (2017) reported that the positive and negative alleles for seed oil and protein contents are often colocalized, and this results in a negative correlation between these two traits (Chao et al., 2017). However, independent QTL for these two traits has also been reported by Zhao et al. (2006). Based on the results from the present study, it can be inferred that the genomic region of *B. oleracea* introgressed into *B. napus* carry QTL for both oil and protein contents and the alleles of this diploid progenitor species can positively contribute to these two seed constituents. These two seed constituents receive their precursor from the same carbon pool through the primary carbohydrate metabolic pathway during the development of the embryo (reviewed in Chao et al., 2017); this competition for precursor can result a negative correlation between these two traits. However, based on data from the present study, it is apparent that the genes from this genomic region might have contributed independently to the biosynthesis of these two seed constituents; competition for the precursor might be one of the reasons for the low additive effect of these oil and protein QTL that we observed in the present study.

Quantitative trait loci mapping of seed GSL content in *B. napus* has also been conducted by several researchers—Basunanda et al. (2007), Fu et al. (2015), Gajardo et al. (2015), He, Fu, Hu, Wei, and Qian (2018), Howell et al. (2003), Liu et al. (2016a), Qian, Qian, and Snowdon (2014), Rücker and Röbbelen (1994), Schatzki et al. (2014), Toroser et al. (1995), Uzunova et al. (1995), Zhao and Meng (2003)—and in *B. oleracea* by Sotelo, Soengas, Velasco, Rodríguez, and Cartea (2014). These studies detected QTL mostly from the C genome chromosomes, where the QTL from C9 has been reported in multiple studies (Basunanda et al., 2007; Gajardo et al., 2015; Liu et al., 2016a; Qian et al., 2014; Schatzki et al., 2014; Sotelo et al., 2014). For example, Schatzki et al. (2014) detected a major QTL on C9 of *B. napus*; this QTL is flanked by the SSR markers CB10357a and Ol10D08/Ol10C10/CB10345 located at 33.5 cM of the 117‐cM linkage map constructed by using SSR and amplified fragment length polymorphism markers. Sotelo et al. (2014) identified a QTL affecting total GSL content in *B. oleracea* at 64.9 cM position of the 71.0‐cM map constructed by using restriction fragment length polymorphism and SSR markers. In the present study, in addition to SNP markers, we also used 55 SSR markers for construction of the genetic linkage map of the C genome; however, position of the QTL markers from these two studies could not be aligned because of a lack of common markers. Nonetheless, based on relative position of the QTL, it is evident that the C9 QTL detected by Sotelo et al. (2014) located at the lower part (64.9 cM of 71.0‐cM map) of the linkage group and the QTL that we detected in our study also positioned at the lower part (125.1–137.2 cM of the 146.8‐cM map) of the C9 chromosome. Thus, based on relative position of the QTL, it can be inferred that the C9 QTL detected by Sotelo et al. (2014) and the QTL detected in the present study could be the same. Basunanda et al. (2007) identified a major QTL on C9 by using a *B. napus* linkage map constructed by using amplified fragment length polymorphism and SSR markers; however, the flanking SSR markers detected by this research group were not included in our map. Gajardo et al. (2015) detected four SNP markers—SNP650, SNP1480, SNP1506, and SNP2521—of C9 associated with total GSL content. A BLASTn search of the sequences provided by Gajardo et al. (2015) showed that they are located at 8.0, 3.9, 16.9, and 1.1 Mb, respectively. In the present study, by using a population segregating for a major QTL for seed GSL content, we identified a QTL on C9 at about 7–8 Mb (Supplemental Table S8); this provides a strong evidence that a QTL for seed GSL is present at this position. In contrast, Körber et al. (2016) found two SNP at 1.7 and 1.8 Mb of C9 affecting GSL content in *B. napus*. Thus, it is possible that the chromosome C9 carries more than one QTL affecting total GSL content in *B. napus*. We also detected a second QTL affecting seed GSL content at about 3–4 Mb of C9 through association mapping (Supplemental Table S8); however, this QTL could not be detected through composite interval mapping.

Epistatic interaction might play a role in the genetic control of seed oil and GSL contents (Würschum et al., 2013; Zhao et al., 2005, 2006); however, we did not find any epistatic interaction for these two seed quality traits that might be a result of small genetic difference between the two parents used in this study. Based on pedigree, the two parents Hi‐Q and RIL‐144 differ only for a part of the C genome introgressed from the C genome of *B. oleracea* var. *alboglabra*.

The bimodal distribution of the DH population for seed GSL content and the involvement of a major QTL in the observed variation for this trait allowed us to partition the population for this QTL alleles based on the flanking markers and evaluate the effect of this QTL on other seed quality and agronomic traits including seed yield. As expected, these two groups of DH lines were significantly different for seed GSL content, which further confirmed the reliability of the effect of this QTL (Figure 1; Table 3). The lack of significant difference between these two groups of DH lines for seed yield suggests that this QTL did not exert any significant effects on yield. This is also evident from our previous report (Rahman et al., 2017) that no QTL for seed yield could be detected in the same genomic region of C9 affecting seed GSL content. This GSL QTL also did not exert any significant effects on days to flowering and seed oil content; however, it showed a significant effect on seed protein content, where the low GSL group had significantly greater protein content than the high GSL group. It is well known that seed GSLs are biosynthesized from amino acids (Halkier & Gershenzon, 2006). In this regard, it can be assumed that because of a genetic block, a relatively small amount of the precursor amino acids are used for the biosynthesis of GSL in the low GSL lines, and this might have resulted in the availability of a greater amount of these amino acids for the biosynthesis of seed protein in these lines. *Brassica napu*s seed contain primarily aliphatic GSLs biosynthesized from methionine and indole GSLs biosynthesized from tryptophan (Halkier & Gershenzon, 2006). Indole GSL content in the high GSL line is, generally, a couple of micromoles greater than the content in the low GSL lines (Sang & Salisbury, 1988); thus, the genetic difference between the low and high GSL lines is primarily a result of the difference in aliphatic GSLs biosynthesized from methionine. In this regard, the low and high GSL DH lines might differ primarily for the content of aliphatic GSL. Whether these two groups of the DH lines with a significant difference in the content of seed protein also differ for amino acid composition, especially for the content of methionine, needs further investigation. The greater protein content in the low GSL lines could also be a result of colocalization of QTL for GSL and protein contents including the contents of cruciferin and napin proteins, as has been reported by Schatzki et al. (2014) in winter oilseed rape. On the other hand, the lack of the effect of the C9 GSL QTL on seed yield was not surprising given that GSLs are biosynthesized from amino acids while the seed biomass is primarily the product of the genes involved in the biosynthesis of all seed constituents including oil, protein, carbohydrate, fiber, and minerals.

## 5 CONCLUSIONS

In conclusion, by using a mapping population carrying the genome content of *B. oleracea*, we identified a QTL affecting seed oil content on C5 and a QTL on C9 affecting seed GSL content. These two QTL were not affected by field environmental conditions and could be detected in all nine trials. The C5 QTL allele of *B. oleracea* in homozygous condition increased ∼1% for oil and 0.3% for protein, while the C9 QTL allele of *B. oleracea* in homozygous condition increased ∼15.6 μmol GSL g^−1^ seed in *B. napus*. The C9 QTL also showed a significant effect on seed protein content, where the lines carrying the low GSL allele of canola in homozygous condition had ∼0.5% greater protein than the lines carrying the high GSL allele. However, the C5 and C9 QTL did not exert any significant effects on seed yield or days to flowering. Thus, the genomic regions and molecular markers identified in this study should be useful in molecular breeding of the seed quality traits in *B. napus*. Also, the results from this study demonstrates that desirable allele for seed quality traits can be found in *B. oleracea* for use in the breeding of oilseed rape.

## AUTHOR CONTRIBUTION STATEMENT

HR conceived the research, designed the experiments and wrote the manuscript, and BK analyzed all data including construction of the linkage map. Both HR and BK conducted the experiments and investigated the phenotypes, as well as reviewed and approved this submission.

## CONFLICT OF INTEREST DISCLOSURE

Authors declare that they have no conflict of interest.

## ETHICAL STANDARD

Research conducted for this manuscript complies with the ethical rules applicable for this journal, as stated in the Instructions for Authors

## Supporting information



Supporting information

Supporting information
