# Supplementary material for: Mapping of seed quality traits in the C genome of Brassica napus by using a population carrying genome content of B. oleracea and their effect on other traits
Source: Plant Genome. 2021 Apr 5;14(2):e20078. doi: 10.1002/tpg2.20078 (PMC12807213; doi:10.1002/tpg2.20078)
Supplement: Supplementary file 1 [file TPG2-14-e20078-s002.docx]

QTL Mapping of Seed Quality Traits in the C Genome of *Brassica napus* by Using a Population Carrying Genome Content of *B. oleracea* and Their Effect on Other Traits

**The Plant Genome**

**Habibur Rahman*, and Berisso Kebede**

Department of Agricultural, Food and Nutritional Science, University of Alberta, Edmonton, AB T6G 2P5, Canada

Number of pages = 13

Number of Supplemental Figures = 3

Number of Supplemental Tables = 9 + 3 = 12 (Supplemental Tables S10-S12 included in a separate Excel file)

Supplemental Figure S1. Frequency distributions of the doubled haploid (DH) lines of the Hi-Q × RIL-144 cross of *Brassica napus* grown in nine trials at St. Albert, South Campus, Killam and Neapolis in Alberta for seed oil content (% of whole seed).

Supplemental Figure S2. Frequency distributions of the doubled haploid (DH) lines of the Hi-Q × RIL-144 cross of *Brassica napus* grown in nine trials at St. Albert, South Campus, Killam and Neapolis in Alberta for seed protein content (% of whole seed).

Supplemental Figure S3. Frequency distributions of the doubled haploid (DH) lines of the Hi-Q × RIL-144 cross of *Brassica napus* grown in nine trials at St. Albert, South Campus, Killam and Neapolis in Alberta for seed glucosinolate content (µmol g^-1^ seed).

Supplemental Table S1. Descriptive statistics for seed oil content (% whole seed) of the *Brassica napus* parents Hi-Q and RIL-144 (carrying the C genome content introgressed from *B. oleracea* var. *alboglabra*) and the doubled haploid (DH) population derived from Hi-Q × RIL-144 cross.

| Location & year | Population | Mean ± SD | Range |
| --- | --- | --- | --- |
| St. Albert 2011 | DH lines | 43.7 ± 1.4 | 41 - 47 |
|  | Hi-Q | 47.3 ± 1.2 | 46 - 50 |
|  | RIL144 | - | - |
|  | Peace | 44.6 ± 0.5 | 44 - 45 |
| South Campus 2011 | DH lines | 43.3 ± 1.2 | 41 - 46 |
|  | Hi-Q | 47.0 ± 0.0 | 47 - 47 |
|  | RIL144 | 40.3 ± 0.5 | 40 - 41 |
|  | Peace | 44.5 ± 1.8 | 43 - 46 |
| St. Albert 2012 | DH lines | 43.0 ± 1.3 | 40 - 46 |
|  | Hi-Q | 45.0 ± 0.5 | 44 - 47 |
|  | RIL144 | 40.5 ± 0.9 | 39 - 42 |
|  | Peace | 43.8 ± 0.8 | 43 - 45 |
| South Campus 2012 | DH lines | 40.6 ± 1.1 | 39 - 44 |
|  | Hi-Q | 42.6 ± 0.8 | 42 - 43 |
|  | RIL144 | 38.7 ± 0.7 | 38 - 40 |
|  | Peace | 42.0 ± 0.0 | 42 - 42 |
| Killam 2013 | DH lines | 48.4 ± 1.6 | 45 - 52 |
|  | Hi-Q | 49.4 ± 0.2 | 49 - 50 |
|  | RIL144 | 46.3 ± 0.7 | 45 - 47 |
|  | Peace | 49.8 ± 0.8 | 49 - 50 |
| St. Albert 2013 | DH lines | 43.3 ± 1.3 | 40 - 46 |
|  | Hi-Q | 45.3 ± 1.3 | 44 - 46 |
|  | RIL144 | 40.3 ± 1.3 | 39 - 41 |
|  | Peace | 43.9 ± 0.6 | 43 - 45 |
| South Campus 2014 | DH lines | 45.9 ± 1.5 | 38 - 46 |
|  | Hi-Q | 44.2 ± 0.9 | 43 - 46 |
|  | RIL144 | 41.0 ± 0.9 | 39 - 44 |
|  | Peace | 43.5 ± 0.7 | 43 - 44 |
| Neapolis 2014 | DH lines | 46.0 ± 1.8 | 40 - 50 |
|  | Hi-Q | 46.1 ± 0.7 | 46 - 50 |
|  | RIL144 | 42.9 ± 0.7 | 42 - 44 |
|  | Peace | 48.1 ± 0.9 | 46 - 50 |
| St. Albert 2014 | DH lines | 42.7 ± 1.6 | 39 - 46 |
|  | Hi-Q | 46.1 ± 1.2 | 44 - 48 |
|  | RIL144 | 39.2 ± 0.6 | 38 - 40 |
|  | Peace | 43.1 ± 1.7 | 42 - 45 |
| Pooled mean | DH lines | 44.1 ± 1.4 | 40 -46 |
|  | Hi-Q | 45.9 ± 1.1 | 46 -47 |
|  | RIL144 | 41.2 ± 1.2 | 40 -42 |
|  | Peace | 44.7 ± 1.0 | 44 -45 |

## Supplemental Table S2. Statistical analysis with the 92 doubled haploid (DH) lines, derived from crossing of Hi-Q and RIL-144 (carrying the C genome content introgressed from *B. oleracea* var. *alboglabra*), and tested in nine yield trials in 2011 to 2015 in Alberta for seed oil content (% whole seed).

## (a) Analysis of variance (ANOVA).

| Effect | Num DF | Den DF | F Value | Pr > F |
| --- | --- | --- | --- | --- |
| Genotype | 91 | 1073 | 253.24 | <0.0001 |
| Trials | 8 | 1073 | 167.92 | <0.0001 |
| Genotype × Trials | 728 | 1073 | 8.19 | <0.0001 |

(b) Spearman correlation of the doubled haploid (DH) lines between yield trials for oil content.

|  | SC2011 | SA2012 | SC2012 | KL2013 | SA2013 | SC2014 | NP2014 | SA2015 | LMOil |
| --- | --- | --- | --- | --- | --- | --- | --- | --- | --- |
| SA2011 | 0.64*** | 0.65*** | 0.41*** | 0.34** | 0.35*** | 0.44*** | 0.45*** | 0.45*** | 0.67*** |
| SC2011 |  | 0.7*** | 0.48*** | 0.39*** | 0.37*** | 0.47*** | 0.38*** | 0.34** | 0.65*** |
| SA2012 |  |  | 0.55*** | 0.46*** | 0.44*** | 0.56*** | 0.52*** | 0.46*** | 0.77*** |
| SC2012 |  |  |  | 0.24* | 0.21 | 0.27* | 0.33** | 0.26* | 0.50*** |
| KL2013 |  |  |  |  | 0.75*** | 0.46*** | 0.45*** | 0.36*** | 0.69*** |
| SA2013 |  |  |  |  |  | 0.40*** | 0.33** | 0.40*** | 0.63*** |
| SC2014 |  |  |  |  |  |  | 0.73*** | 0.72*** | 0.83*** |
| NP2014 |  |  |  |  |  |  |  | 0.69*** | 0.82*** |
| SA2015 |  |  |  |  |  |  |  |  | 0.79*** |

*, ** and *** significant at *p* 0.05, 0.01 and ≤ 0.001 respectively

SC = South Campus, SA = St. Albert, KL = Killam, NP = Neapolis, LMOil=Least Square Means of Oil

Supplemental Table S3. Descriptive statistics for seed protein content (% whole seed) of the *Brassica napus* parents Hi-Q and RIL-144 (carrying the C genome content introgressed from *B. oleracea* var. *alboglabra*) and the doubled haploid (DH) population derived from Hi-Q × RIL-144 cross.

| Location and year | Materials | Mean ± SD | Range |
| --- | --- | --- | --- |
| St. Albert 2011 | DH lines | 26.5 ± 0.9 | 24 - 29 |
|  | Hi-Q | 25.4 ± 0.9 | 24 - 26 |
|  | RIL144 | - | - |
|  | Peace | 27.6 ± 0.4 | 27 - 28 |
| South Campus 2011 | DH lines | 27.8 ± 0.7 | 26 - 30 |
|  | Hi-Q | 27.1 ± 0.6 | 26 - 28 |
|  | RIL144 | 28.2 ± 0.6 | 28 - 29 |
|  | Peace | 27.8 ± 0.8 | 26 - 30 |
| St. Albert 2012 | DH lines | 25.5 ± 0.9 | 24 - 28 |
|  | Hi-Q | 25.9 ± 1.3 | 24 - 27 |
|  | RIL144 | 25.9 ± 1.7 | 25 - 27 |
|  | Peace | 26.5 ± 0.7 | 25 - 27 |
| South Campus 2012 | DH lines | 27.7 ± 0.8 | 26 - 29 |
|  | Hi-Q | 27.7 ± 0.5 | 27 - 29 |
|  | RIL144 | 28.9 ± 0.9 | 28 - 30 |
|  | Peace |  |  |
| Killam 2013 | DH lines | 21.5 ± 1.0 | 20 - 24 |
|  | Hi-Q | 21.8 ± 0.7 | 21 - 22 |
|  | RIL144 | 22.0 ± 1.9 | 20 - 23 |
|  | Peace | 22.3 ± 0.8 | 21 - 23 |
| St. Albert 2013 | DH lines | 26.1 ± 0.8 | 24 - 28 |
|  | Hi-Q | 26.3 ± 1.2 | 25 - 27 |
|  | RIL144 | 27.9 ± 0.7 | 27 - 29 |
|  | Peace | - | - |
| South Campus 2014 | DH lines | 26.8 ± 0.9 | 25 - 29 |
|  | Hi-Q | 27.5 ± 1.6 | 26 - 29 |
|  | RIL144 | 26.3 ± 1.9 | 24 - 28 |
|  | Peace | 28.4 ± 0.7 | 28 - 29 |
| Neapolis 2014 | DH lines | 23.9 ± 1.3 | 20 - 27 |
|  | Hi-Q | 26.1 ± 0.9 | 25 - 27 |
|  | RIL144 | 24.3 ± 0.7 | 23 - 26 |
|  | Peace | 24.0 ± 1.9 | 22 - 26 |
| St. Albert 2015 | DH lines | 27.1 ± 1.1 | 25 - 30 |
|  | Hi-Q | 26.2 ± 0.9 | 24 - 29 |
|  | RIL144 | 28.5 ± 0.6 | 28 - 30 |
|  | Peace | 25.4 ± 0.7 | 23 - 27 |
| Pooled mean | DH lines | 25.1 ± 0.6 | 24 - 27 |
|  | Hi-Q | 25.9 ± 1.2 | 21 - 29 |
|  | RIL144 | 26.2 ± 0.6 | 21 - 30 |
|  | Peace | 25.7 ± 1.0 | 22 - 29 |

## Supplemental Table S4. Statistical analysis with the 92 doubled haploid (DH) lines, derived from crossing of Hi-Q and RIL-144 (carrying the C genome content introgressed from *B. oleracea* var. *alboglabra*), and tested in nine yield trials in 2011 to 2015 in Alberta for seed protein content (% whole seed).

## (a) Analysis of variance (ANOVA).

| Effect | Num DF | Den DF | F Value | Pr > F |
| --- | --- | --- | --- | --- |
| Genotype | 91 | 1073 | 2.98 | <0.0001 |
| Location | 8 | 1073 | 394.3 | <0.0001 |
| Genotype × Location | 728 | 1073 | 0.81 | 0.99 |

(a) Spearman correlation of the doubled haploid (DH) lines between yield trials for protein content.

|  | SC2011 | SA2012 | SC2012 | KL2013 | SA2013 | SC2014 | NP2014 | SA2015 | LMPro |
| --- | --- | --- | --- | --- | --- | --- | --- | --- | --- |
| SA2011 | 0.67*** | 0.61*** | 0.25* | 0.09 | 0.26* | 0.35** | 0.38*** | 0.38*** | 0.60*** |
| SC2011 |  | 0.64*** | 0.34** | 0.16 | 0.37*** | 0.37*** | 0.30** | 0.27* | 0.58*** |
| SA2012 |  |  | 0.40*** | 0.08 | 0.15 | 0.32** | 0.25* | 0.23* | 0.51*** |
| SC2012 |  |  |  | 0.03 | 0.22* | 0.18 | 0.24* | 0.23* | 0.44*** |
| KL2013 |  |  |  |  | 0.41*** | 0.08 | 0.11 | 0.14 | 0.40*** |
| SA2013 |  |  |  |  |  | 0.15 | 0.12 | 0.40*** | 0.41*** |
| SC2014 |  |  |  |  |  |  | 0.28** | 0.44*** | 0.38*** |
| NP2014 |  |  |  |  |  |  |  | 0.39*** | 0.75*** |
| SA2015 |  |  |  |  |  |  |  |  | 0.67*** |

*, ** and *** significant at *p* 0.05, 0.01 and ≤ 0.001 respectively

SC = South Campus, SA = St. Albert, KL = Killam, NP = Neapolis, LMPro=Least Square Means Protein

Supplemental Table S5. Descriptive statistics for seed glucosinolate content (µmol/g seed) of the *Brassica napus* parents Hi-Q and RIL-144 (carrying the C genome content introgressed from *B. oleracea* var. *alboglabra*) and the doubled haploid (DH) population derived from Hi-Q × RIL-144 cross.

| Location & year | Materials | Mean ± SD | Range |
| --- | --- | --- | --- |
| St. Albert 2011 | DH lines | 27.0 ± 8.6 | 14 - 40 |
|  | Hi-Q | 14.4 ± 2.1 | 12 - 17 |
|  | RIL144 | - | - |
|  | Peace | 16.4 ± 1.0 | 15 - 18 |
| South Campus 2011 | DH lines | 24.4 ± 9.1 | 13 - 42 |
|  | Hi-Q | 15.0 ± 1.3 | 13 - 16 |
|  | RIL144 | 33.2 ± 5.2 | 30 - 37 |
|  | Peace | 16.8 ± 1.1 | 15 - 18 |
| St. Albert 2012 | DH lines | 27.7 ± 8.9 | 14 - 44 |
|  | Hi-Q | 15.5 ± 0.4 | 15 - 16 |
|  | RIL144 | 35.7 ± 0.7 | 35 - 36 |
|  | Peace | 16.6 ± 0.6 | 16 - 18 |
| South Campus 2012 | DH lines | 28.5 ± 8.8 | 16 - 41 |
|  | Hi-Q | 15.1 ± 0.7 | 14 - 17 |
|  | RIL144 | 36.6 ± 0.7 | 36 - 37 |
|  | Peace | 15.7 ± 0.0 | 16 - 16 |
| Killam 2013 | DH lines | 26.0 ± 8.6 | 12 - 40 |
|  | Hi-Q | 13.6 ± 0.4 | 13 - 14 |
|  | RIL144 | 33.2 ± 1.2 | 32 - 34 |
|  | Peace | 18.0 ± 1.2 | 17 - 19 |
| St. Albert 2013 | DH lines | 27.5 ± 8.9 | 13 - 44 |
|  | Hi-Q | 14.4 ± 0.4 | 14 - 15 |
|  | RIL144 | 35.2 ± 0.2 | 35 - 36 |
|  | Peace | 19.0 ± 1.3 | 17 - 20 |
| South Campus 2014 | DH lines | 29.1 ± 10.8 | 12 - 48 |
|  | Hi-Q | 17.7 ± 0.9 | 17 - 18 |
|  | RIL144 | 41.6 ± 4.8 | 36 - 47 |
|  | Peace | 17.7 ± 0.8 | 17- 18 |
| Neapolis 2014 | DH lines | 29.1 ± 10.8 | 14 - 48 |
|  | Hi-Q | 16.7 ± 0.3 | 16 - 17 |
|  | RIL144 | 40.2 ± 2.4 | 39 - 45 |
|  | Peace | 16.9 ± 0.9 | 16 - 18 |
| St. Albert 2015 | DH lines | 29.1 ± 10.8 | 14 - 52 |
|  | Hi-Q | 15.5 ± 1.0 | 14 - 16 |
|  | RIL144 | 48.3 ± 1.2 | 47 - 50 |
|  | Peace | 16.9 ± 0.9 | 16 - 18 |
| Pooled mean | DH lines | 27.6 ± 9.5 | 16 -44 |
|  | Hi-Q | 15.3 ± 0.5 | 15 -16 |
|  | RIL144 | 38.0 ± 1.1 | 37 -40 |
|  | Peace | 17.1 ± 0.9 | 16 -19 |

## Supplemental Table S6. Statistical analysis with the 92 doubled haploid (DH) lines, derived from crossing of Hi-Q and RIL-144 (carrying the C genome content introgressed from *B. oleracea* var. *alboglabra*), and tested in nine yield trials in 2011 to 2015 in Alberta for seed glucosinolate content (µmol/g seed).

## (a) Analysis of variance (ANOVA).

| Effect | Num DF | Den DF | F Value | Pr > F |
| --- | --- | --- | --- | --- |
| Genotype | 91 | 1073 | 9.09 | <0.0001 |
| Location | 8 | 1073 | 439.84 | <0.0001 |
| Genotype × Location | 728 | 1073 | 1.18 | 0.0072 |

(b) Spearman correlation of the doubled haploid (DH) lines between yield trials for glucosinolate content.

|  | SC2011 | SA2012 | SC2012 | KL2013 | SA2013 | SC2014 | NP2014 | SA2015 | LMGLS |
| --- | --- | --- | --- | --- | --- | --- | --- | --- | --- |
| SA2011 | 0.93*** | 0.96*** | 0.96*** | 0.64*** | 0.64*** | 0.87*** | 0.85*** | 0.86*** | 0.94*** |
| SC2011 |  | 0.92*** | 0.92*** | 0.64*** | 0.64*** | 0.82*** | 0.78*** | 0.82*** | 0.90*** |
| SA2012 |  |  | 0.99*** | 0.68*** | 0.69*** | 0.87*** | 0.86*** | 0.87*** | 0.95*** |
| SC2012 |  |  |  | 0.66*** | 0.66*** | 0.87*** | 0.86*** | 0.87*** | 0.95*** |
| KL2013 |  |  |  |  | 0.99*** | 0.58*** | 0.56*** | 0.59*** | 0.73*** |
| SA2013 |  |  |  |  |  | 0.58*** | 0.56*** | 0.58*** | 0.73*** |
| SC2014 |  |  |  |  |  |  | 0.97*** | 0.98*** | 0.96*** |
| NP2014 |  |  |  |  |  |  |  | 0.97*** | 0.95*** |
| SA2015 |  |  |  |  |  |  |  |  | 0.96*** |

*, ** and *** significant at *p* 0.05, 0.01 and ≤ 0.001 respectively

SC = South Campus, SA = St. Albert, KL = Killam, NP = Neapolis, LMGLS= Least Square Means of glucosinoalte

Supplemental Table S7. List of SNP markers from the QTL regions associated with seed oil content in a *Brassica napus* doubled haploid (DH) population derived from crossing of Hi-Q and RIL-144 (carrying the C genome content introgressed from *B. oleracea* var. *alboglabra*)

| Marker name | Chr./cM | Start | | End | | R^2a^ | | Bonferroni- corrected significant threshold | | Allele | |  |
| --- | --- | --- | --- | --- | --- | --- | --- | --- | --- | --- | --- | --- |
| *SNP markers based on genome-wide association study*: | | | | | | | | | | | |  |
| Bn-scaff_17441_3-p91321 | C5 | 31408748 | | 31408869 | | 36.24 | | 6.281 | | T/C | |  |
| **Bn-A05-p22111286** | **C5** | **39985294** | | **39985492** | | **37.24** | | **6.408** | | **A/G** | |  |
| Bn-scaff_17441_1-p558858 | C5 | 40516371 | | 40516491 | | 31.17 | | 5.620 | | T/C | |  |
| Bn-scaff_17441_1-p1039913 | C5 | 40727336 | | 40727456 | | 28.62 | | 5.277 | | A/G | |  |
| **Bn-scaff_17441_1-p1140035** | **C5** | **40835440** | | **40835559** | | **27.06** | | **5.062** | | **A/G** | |  |
| Bn-scaff_17441_1-p1211653 | C5 | 40924813 | | 40924933 | | 27.06 | | 5.062 | | T/C | |  |
| Bn-scaff_17441_3-p54682 | C5 | 41013514 | | 41013620 | | 27.06 | | 5.062 | | A/G | |  |
| Bn-scaff_17441_3-p56937 | C5 | 41015807 | | 41015927 | | 27.17 | | 5.078 | | T/C | |  |
| Bn-scaff_17441_3-p154031 | C5 | 41061897 | | 41062018 | | 24.41 | | 4.693 | | A/G | |  |
| Bn-scaff_17441_3-p156691 | C5 | 41064556 | | 41064677 | | 18.35 | | 3.810 | | T/C | |  |
| Bn-scaff_29028_1-p15505 | C5 | 41554621 | | 41554741 | | 19.87 | | 4.036 | | A/G | |  |
| Bn-A05-p22813805 | C5 | 41717985 | | 41718243 | | 18.14 | | 3.777 | | T/C | |  |
| Bn-scaff_23186_1-p8774 | C5 | 42711516 | | 42711636 | | 19.65 | | 4.003 | | A/G | |  |
| Bn-scaff_20270_1-p1070285 | C5 | 42863600 | | 42863720 | | 19.65 | | 4.003 | | A/G | |  |
| Bn-scaff_20270_1-p1211632 | C5 | 42991334 | | 42991454 | | 18.14 | | 3.777 | | T/C | |  |
| *SNP markers based on composite interval mapping*: |  | |  | |  | |  | |  | |  | |
| **Bn-A05-p22111286** | **C5 / 77.6** | **39985294** | | **39985492** | | **33.6** | |  | | **A/G** | |  |
| **Bn-scaff_17441_1-p1142314** | **C5 / 56.4** | **40837720** | | **40837840** | | **33.6** | |  | | **T/C** | |  |
| Bn-A05-p22813805 | C5 / 72.7 | 41717985 | | 41718243 | | 8.4 | |  | | T/C | |  |

^a^ R^2^ = Percent phenotypic variance explained by the SNP marker

N.B. Markers detected by using both composite interval mapping and GWAS approaches are marked with bold font.

Supplemental Table S8. List of SNP markers from the QTL regions associated with seed glucosinolate content in a *Brassica napus* doubled haploid (DH) population derived from crossing of Hi-Q and RIL-144 (carrying the C genome content introgressed from *B. oleracea* var. *alboglabra*)

| Marker name | Chr./cM | Start | End | R^2a^ | Bonferroni- corrected significant threshold | Allele |
| --- | --- | --- | --- | --- | --- | --- |
| *SNP markers based on genome-wide association study*: |  |  |  |  |  |  |
| Bn-A09-p2730673 | C9 | 2953271 | 2953471 | 66.11 | 9.581 | T/C |
| Bn-scaff_19783_1-p379086 | C9 | 4122694 | 4122887 | 67.93 | 9.776 | A/C |
| **Bn-scaff_17190_1-p237949** | **C9** | **6961870** | **6962170** | **56.00** | **8.448** | **T/C** |
| **Bn-scaff_17297_1-p214513** | **C9** | **6961870** | **6962170** | **42.40** | **6.789** | **A/G** |
| Bn-scaff_17084_1-p851121 | C9 | 8607645 | 8607766 | 35.97 | 5.941 | A/G |
| *SNP/SSR markers based on composite interval mapping*: |  |  |  |  |  |  |
| **Bn-scaff_17297_1-p214513** | **C9 / 132.3** | **6961870** | **6962170** | **60.5** |  | **A/G** |
| **Bn-scaff_17190_1-p237949** | **C9** | **6961870** | **6962170** | **73.8** |  | **T/C** |
| Bn-scaff_17084_1-p851121 | C9 / 125.1 | 8204377 | 8204575 | 73.8 |  | A/G |

^a^ R^2^ = Percent phenotypic variance explained by the SNP marker

N.B. Markers detected by using both composite interval mapping and GWAS approaches are marked with bold font.

## Supplemental Table S9. Effect of the C5 and C9 QTL alleles of Hi-Q and RIL-144 (carrying the C genome content introgressed from *B. oleracea* var. *alboglabra*) of *Brassica napus* in a doubled haploid (DH) population derived from Hi-Q × RIL-144 on seed quality and agronomic traits including seed yield. The DH population tested in nine yield trials in 2011 to 2015 in Alberta and partitioned for the QTL alleles.

| QTL^1^ | Origin of allele | No. DH  lines | Oil  (% seed) | Protein  (% seed) | Glucosinolate  (µmol/g seed) | Days to  flower | Seed yield (kg/ha) |
| --- | --- | --- | --- | --- | --- | --- | --- |
| C5 | Hi-Q | 21 | 43.0 ± 0.2 | 24.9 ± 0.1 | 30.8 ± 2.1 | 48.0 ± 0.3 | 2947.6 ± 45.5 |
|  | RIL-144 | 59 | 44.0 ± 0.1 | 25.2 ± 0.1 | 27.9 ± 1.1 | 47.8 ± 0.2 | 2934.6 ± 32.7 |
| t-test | p-value (Pooled) |  | <0.0001 | 0.0506 | 0.2163 | 0.6771 | 0.8315 |
|  | p-value (Satterthwaite) |  | <0.0001 | 0.0352 | 0.2477 | 0.6629 | 0.8175 |
|  | Significant difference |  | Yes | Yes | No | No | No |
| C9 | Hi-Q | 28 | 44.1 ± 0.2 | 25.3 ± 0.1 | 20.5 ± 0.7 | 48.0 ± 0.2 | 2901.6 ± 52.4 |
|  | RIL-144 | 35 | 43.8 ± 0.2 | 24.8 ± 0.1 | 36.1 ± 0.5 | 48.0 ± 0.2 | 2955.3 ± 33.0 |
| t-test | p-value (Pooled) |  | 0.1665 | 0.0005 | <0.0001 | 0.9085 | 0.3700 |
|  | p-value (Satterthwaite) |  | 0.1735 | 0.0005 | <0.0001 | 0.9084 | 0.3897 |
|  | Significant difference |  | No | Yes | Yes | No | No |

^1^ C5 QTL associated with seed oil content and C9 QTL associated with seed glucosinolate content. These two QTL detected in all nine field trials.
